# Machining of Triangular Holes in D2 Steel by the Use of Non-Conventional Electrodes in Die-Sinking Electric Discharge Machining

**DOI:** 10.3390/ma16103865

**Published:** 2023-05-20

**Authors:** Madiha Rafaqat, Nadeem Ahmad Mufti, Muhammad Qaiser Saleem, Naveed Ahmed, Ateekh Ur Rehman, Muhammad Asad Ali

**Affiliations:** 1 Department of Industrial and Manufacturing Engineering, University of Engineering and Technology, Lahore 54890, Pakistan; namufti@uet.edu.pk (N.A.M.); qaiser@uet.edu.pk (M.Q.S.); asad.ali@uet.edu.pk (M.A.A.); 2 Department of Industrial Engineering, College of Engineering and Architecture, Al-Yamamah University, Riyadh 11512, Saudi Arabia; n_ahmed@yu.edu.sa; 3 Department of Industrial Engineering, College of Engineering, King Saud University, Riyadh 11421, Saudi Arabia; arehman@ksu.edu.sa

**Keywords:** electric discharge machining (EDM), non-conventional electrode, relief angle, D2 Steel, taper angle, surface roughness, machining, material removal rate

## Abstract

Electric discharge machining is relatively a slow process in terms of machining time and material removal rate. The presence of overcut and the hole taper angle caused by the excessive tool wear are other challenges in the electric discharge machining die-sinking process. The areas of focus to solve these challenges in the performance of electric discharge machines include increasing the rate of material removal, decreasing the rate of tool wear, and reducing the rate of hole taper angle and overcut. Triangular cross-sectional through-holes have been produced in D2 steel through die-sinking electric discharge machining (EDM). Conventionally, the electrode with uniform triangular cross-section throughout the electrode length is used to machine triangular holes. In this study, new designs of electrodes (non-conventional designs) are employed by introducing circular relief angles. For material removal rate (MRR), tool wear rate (TWR), overcut, taper angle, and surface roughness of the machined holes, the machining performance of conventional and unconventional electrode designs is compared. A significant improvement in MRR (32.6% increase) has been achieved by using non-conventional electrode designs. Similarly, the hole quality resulted by non-conventional electrodes is way better than hole quality corresponding to conventional electrode designs, especially in terms of overcut and hole taper angle. A reduction of 20.6% in overcut and a reduction of 72.5% in taper angle can be achieved through newly designed electrodes. Finally, one electrode design has been selected (electrode with 20 degree relief angle) as the most appropriate electrode resulting in better EDM performance in terms of MRR, TWR, overcut, taper angle, and surface roughness of triangular holes.

## 1. Introduction

Electric discharge machining (EDM) is among the oldest non-conventional machining processes. The literature is heavily dense on various domains of EDM [1]. EDM is one of the very commonly used process to cut and machine hard materials. EDM die-sinking is noted for its lengthy machining processes and low rate of material removal. Significant tool wear rates, taperness in the sidewall of the machined features, and significant surface roughness are additional difficulties with EDM die-sinking [2]. Numerous approaches have been developed by researchers to deal with these problems. These initiatives are evaluated in this literature review.

The effect of EDM process parameters on the EDM performance measures has been widely researched. For instance, the spark voltage and electrode polarity significantly contributes towards the improvement in material removal rate [3]. It is important to take into account process parameter fluctuation when determining the causes and effects of MRR and TWR, such as discharge peak current and pulse duration [4]. The discharge energy level varies depending on how the EDM process parameters are changed. The size and radius of craters are directly impacted once a difference in discharge energy is generated during experiments. This immediately influences the MRR and surface roughness of the machined areas [5]. Thus, planned preparation of molds with changing surface roughness, variations in discharge current and pulse duration, have a direct impact on the surface roughness of the machined surface [6]. Similarly, inter-electrode gap is another reported parameter affecting the MRR and surface roughness. In order to achieve the high MRR, inter-electrode gap should be reduced [7]. In this regard, the optimization of process parameters is the key to achieve optimized output responses, such as MRR, TWR, overcut, surface roughness, etc. [8]. Jampana et al. [9] proposed the optimized set of EDM parameters, through the use of grey relational analysis, to achieve high MRR with low surface roughness for EDM of stainless steel 630. Likewise, parametric combination for EDM of magnesium is reported in [10]. Similarly, the most cogent parametric combination has been proposed by Sharma et al. [11] to machine nickel alloys 625 and 718 for the optimized MRR, TWR, surface roughness, and overcut. Optimization of EDM parameters for different materials have also been reported, such as for AISI 329 steel [12], SAE 304 [13], and D2-die steel [14].

The use of different electrode materials can be seen in many studies where the effect of electrode material on machining has been investigated [10]. Every type of electrode material offers different rate of material erosion in EDM. Therefore, the proper and suitable selection of electrode material is important to increase the material removal rate. Ahmed et al. [15] used four different materials of electrodes and found graphite as the suitable material for machining titanium alloy with high MRR and low surface roughness. EDM process changes the structural properties of the workpiece, especially at the neighboring areas of the electric sparks. The micro-structural changes depends on the material of the electrode [16]. Some noticeable changes caused by the EDM process includes recast layers, pores, micro-cracks, and craters on the machined surfaces [17]. The presence of residual stresses is evident that leads to crack propagation at micro scale in steel [18]. The EDM process parameters play important roles in inducing residual stresses. Current and pulse duration are the key parameters that contribute towards the tensile residual stresses while electric discharge aids in machining of steel. Addition of foreign materials (powders of different materials) in the conventional dielectric fluid is another commonly practiced norm in the field of electric discharge machining. This variant is called powder mixed electric discharge machining (PMEDM). There are two main reasons behind the popularity of PMEDM. One reason is to improve the MRR and the other reason is to achieve the surface modifications at the machined areas. Surface modifications are mainly investigated for biomedical applications, such as biomedical implants [19].

The use of different di-electric fluids is another direction where researchers have investigated the effect of fluid type on EDM responses, especially the effect of dielectrics on MRR and surface roughness. For instance, the EDM oil, castor oil and hydrocarbon oil are used in [10], where EDM of magnesium is carried out. An improvement of 5% in MRR has been reported when the machining is conducted in the presence of castor oil as compared to EDM oil. It is also evident that the use of bio-dielectrics in EDM is becoming more widespread not only to increase MRR but also to advance the cause of sustainability and becoming green in the machining industry [20]. In a different study [21], non-edible vegetable oils were utilized in the EDM process, and a comparison analysis showed that the use of these oils resulted in superior MRR, lower tool wear, and lower surface roughness. The MRR of magnetic field-assisted micro-EDM is 22% higher than non-magnetic field-assisted micro-EDM [22]. Near-dry electric discharge machining is another reported method to improve the EDM machining performance measures [23]. Bhatt et al. [24] combined PMEDM with the assistance of magnetic field and achieved improved MRR and surface roughness. Another approach to improve the EDM performance is reported in [25] wherein novel electrodes are used to machine blind circular holes in mild steel. Polymer-based ABS electrodes are electroplated with copper to ensure the conductivity of polymeric electrodes. The dimensional accuracy comparison is performed in terms of overcut (change in hole diameters) and hole depth. It has been concluded that the performance of novel electrodes is better than the conventional copper electrodes in terms of hole accuracy.

Triangular shaped electrode having uniform cross-section throughout its entire length is conventionally used to machine triangular holes. In this study, non-conventional electrode designs are introduced to machine triangular holes in D2 steel through die-sinking EDM. A concept of circular relief angle is utilized to design the upper body of the new electrodes with a triangular face. The performance of non-conventional electrodes is compared with that of conventional electrode in terms of five output responses including material removal rate (MRR), tool wear rate (TWR), overcut, hole taper angle, and surface roughness. Machining results reveal that significant improvement can be achieved in machining responses by using non-conventional electrodes. For instance, an improvement of 32.6% in MRR can be realized with the help of electrodes employed in this study. Likewise, the overcut and the hole taper angle can be reduced by 20.6% and 72.5%, respectively, if the non-conventional electrodes are used in place of conventional electrode.

## 2. Materials and Methods

**Figure 1:**
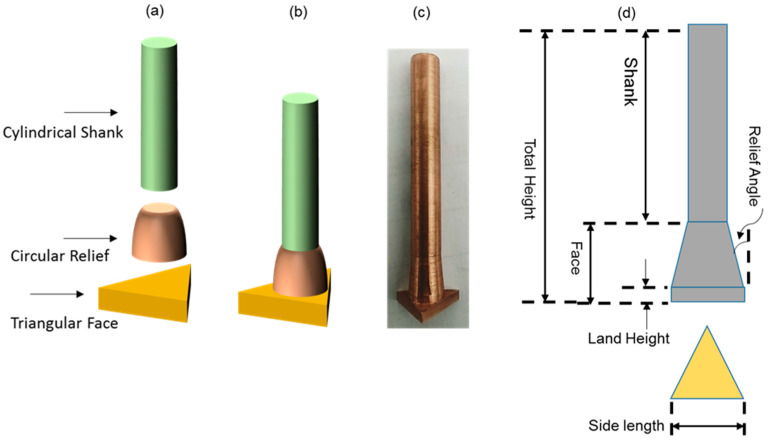
Nonconventional electrodes: (**a**) individual segments, (**b**) assembled 3D schematic, (**c**) actual non-conventional electrode, and (**d**) 2D schematic for different lengths and heights.

D2-grade of steel is among the widely used materials in die-making industry. Different parts of dies and molds consists of through-holes of circular and non-circular cross-sections. With reference to machining processes in making dies and molds, die-sinking electric discharge machining (EDM) is frequently involved. Therefore, in this research through-holes of triangular cross-section are produced in D2-grade of steel by electric discharge machining. Conventional tools consist of uniform cross-section along the length of the tool. Here, in this research, tools are designed in such a way that the cross-section of the tool along its length is different than its cross-section at the workpiece facing end. Six different designs are developed for tools (non-conventional designs) and their machining performance is compared with the conventional tool. The schematic diagrams (2D and 3D) and actual fabricated non-conventional tools are shown in Figure 1. The electrode consists of three segments named as cylindrical shank, circular relief, and triangular face, as labelled in Figure 1. Dimensions of different segments of each of the design elements are provided in Table 1.

To achieve consistency and repeatability in tool preparation, only one copper rod per tool was used. On the same workpiece, machining runs were carried out similarly. Machining settings were maintained at fixed values. Generally, when the levels of the machining parameters are changed during experimentation, the machining results are influenced under the change of machining parameters. Therefore, in order to ensure that the machining results reflect only the effect of tool designs on the EDM performance measures, all the EDM process parameters and other machining conditions were maintained at the same settings during each experiment of the present research. Machining conditions and other constant parameters of tools and holes are listed in Table 2. The ranges of parametric values for EDM using a copper electrode are drawn from the literature, which is given in the introduction section. Since the EDM settings are maintained at the same level throughout the experiment, the effect of the EDM parameters is consistent. Every experiment shows how tool design has an impact. Material removal rate (MRR), tool wear rate (TWR), side over-cut, taper angle, and the surface roughness of the machined holes are considered as the EDM performance characteristics.

With reference to measurements of the above stated responses, data recording and measurements were carefully carried out. Weighing method of measurement was used to calculate the MRR and TWR. Weights of tools and the workpiece were recorded before and after machining with digital weighing balance having 1 g weighing accuracy. The difference in weights was divided by the density of D2 steel and machining time to get material removal rate (MRR). The unit of density was taken as g/cm^3^. Then, cm^3^ were converted to µm^3^. Similarly, TWR was calculated. For tool wear rate, the density of copper was computed in the calculations. The calculation of MRR is given below in the form of Equation (1).

(1) $$MRR=\frac{{∆W}_{D2}}{\left(\rho_{D2}\times T_{m}\right)}\;\mathrm{Or}\;MRR=\frac{V_{m}}{T_{m}}$$

where,

$${∆W}_{D2}=W_{before\;machining}-W_{after\;machining}$$

$$\rho_{D2}=Density\;of\;D2\;steel$$

$$V_{m}=volume\;of\;material\;removed({\mu m}^{3})$$

$$V_{m}=\frac{{∆W}_{D2}}{\rho_{D2}}$$

$$T_{m}=Machining\;time$$

The tool wear calculations were also made in the similar way as that of material removal calculations. Equation (2) represents the formula for tool wear rate.

(2) $$TWR=\frac{{∆W}_{Cu}}{\left(\rho_{Cu}\times T_{m}\right)}\;\mathrm{Or}\;TWR=\frac{V_{m}}{T_{m}}$$

where,

$${∆W}_{Cu}=W_{before\;machining}-W_{after\;machining}$$

$$\rho_{Cu}=Density\;of\;Copper$$

$$V_{m}=volume\;of\;material\;removed({\mu m}^{3})$$

$$V_{m}=\frac{{∆W}_{Cu}}{\rho_{Cu}}$$

$$T_{m}=Machining\;time$$

With the help of the coordinate measuring machine (CE-450DV, CHIEN WEI Precise Technology Co., LTD., Taipei, Taiwan), having 1 µm accuracy, the hole dimensions were recorded at six points and the average was taken. This average value was used to get the overcut, which is the difference between the tool size and the hole size. Likewise, the recording from the CMM was used to calculate taper angle of machined holes. Surface roughness measurements were taken with Surtronic surface roughness meter. An evaluation length of 3 mm was taken to record roughness values. A fixture was designed to hold the workpiece at a firm place and to ensure correctness of each measurement. Three-point measurements were recorded and average R_a_ values are reported.

## 3. Results and Discussion

The machining experiments were performed using seven different electrodes, including conventional designed electrode and relief angled electrodes. The conventional electrode is termed as C0 whereas the electrodes with different relief angles are alpha-numerically termed as CR5, CR10, CR20, CR30, CR40, and CR90. Here, CR represents the circular relief while the numbers reflect the relief angle designed for the tool. Relief angles were systematically designed from 5 degree to 40 degree to investigate the effect of relief angled designed tools. In this way, further increments to relief angle could be 50, 60, 70, 80, and 90. It was decided that five relief angles (5, 10, 20, 30, and 40 degrees) would be enough to establish the trend therefore further increments to relief angles were omitted in the design of experiments. However, in order to see whether the exceptionally enlarged relief angle has some significant impact on the important performance measures or not, the relief angle of 90 degree was taken as a test.

The experimental and measurements results are provided in Table 3. The response measures achieved after machining the holes through conventional electrode design is considered as the reference of comparison. Machining responses associated with each electrode design (relief angle) are evaluated and each response is compared with the performance measure associated with the conventional electrode.

### 3.1. Effect of Tool Designs on Material Removal Rate

**Figure 2:**
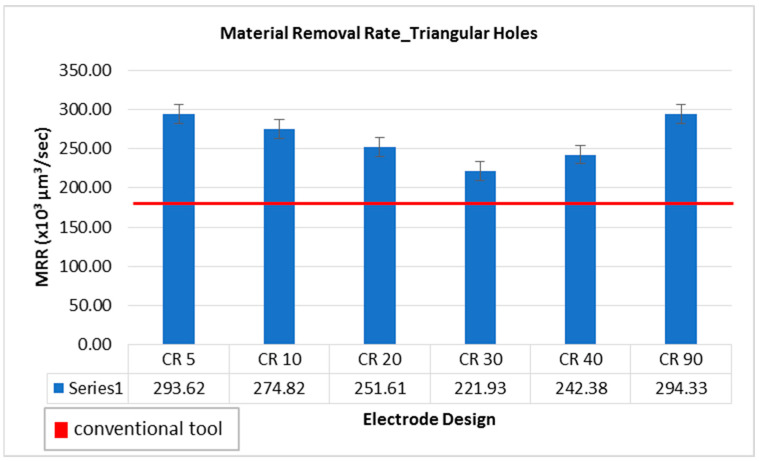
Material removal rate while machining triangular holes with different electrode designs.

In the analysis of the material removal rate (MRR) a multiplication factor of 10^6^ µm^3^/s is common in all cases of machining results. Hence, the MRR achieved using conventional electrode (uniform triangular cross-section throughout the length of electrode) is 221.70 × 10^6^ µm^3^/s. Comparing the MRR generated using non-conventional electrodes, each design resulted in significantly higher MRR as compared to conventional electrodes. The graphical representation is shown in Figure 2. The red horizontal line is a reference line corresponding to MRR of conventional electrode (221.70 × 10^6^ µm^3^/s). Considering the trend of non-conventional electrode designs, the MRR is relatively decreased by the increase in relief angle from 5 degree to 30 degree. With respect to conventional electrode, the MRR associated with each of the non-conventional electrodes is significantly higher. However, 30 to 90 degree relief angles machined more amount of material, increasing the MRR to 294.33 × 10^6^ µm^3^/s. The contribution of relief angles towards this improvement in MRR is mainly because of the reduction in tool-workpiece interacting area while sparking undergoes. In the case of conventional design, due to uniform cross-sectional area of the conventional electrode throughout its length, the tool-workpiece interacting area continuously increases as the machining of hole is progressed. The result is longer machining time and smaller amounts of material removal. Because the erosion is carried out by electrode surfaces close to the substrate surfaces, the phenomenon behind the shorter erosion time and higher material removal rate is thought to be a different sparking behavior in the case of altered tool designs as opposed to sparking offered by conventional designs. Electric discharges can only occur between the electrode’s bottom face and the work sample’s top surface. However, when the hole is made, sparking happens at two locations: the tool’s bottom face and the perimeter of the tool’s segment penetrating the partially formed hole. The typical cylindrical tool design features vertical straight walls along the length of it (face and shank diameters are equal). As a result, in the case of traditional design, the sparking area steadily expanded (D_C_). Nevertheless, the design with relief angle (D_CRL_) permits electric discharges to be created only at the tool’s footing cutting end and land area. It remains consistent until the entire hole is created. The existence of a relief angle inhibits the occurrence of peripheral sparking. The inter-electrode gap between the shank and the inner wall of the hole becomes so large in the presence of relief angle that sparking does not occur. As a result, the same input energy is focused onto the tool’s bottom face, resulting in high energy density. Consequently, the machining time is significantly shortened, and the sparking concentrated area further deteriorates. Therefore, non-conventional electrodes offer variation in their cross-sections at different parts of the electrodes. Hence, the interacting or machining area does not progressively increase. The concept of land (the facing part of the non-conventional electrodes with constant thickness of 2 mm) ensures that the interaction or discharge area remains constant as the machining is progressed until the formation of the through-hole. The electrodes with relief angle of 5 and 90 degree resulted in the highest values of MRR 293.62 × 10^6^ µm^3^/s and 294.33 × 10^6^ µm^3^/s, respectively. Since achieving high MRR is a very common requirement of the EDM process, it can be stated that using non-conventional electrodes results in an improvement of 32.76% in material removal rate.

### 3.2. Effect of Tool Designs on Tool Wear Rate

**Figure 3:**
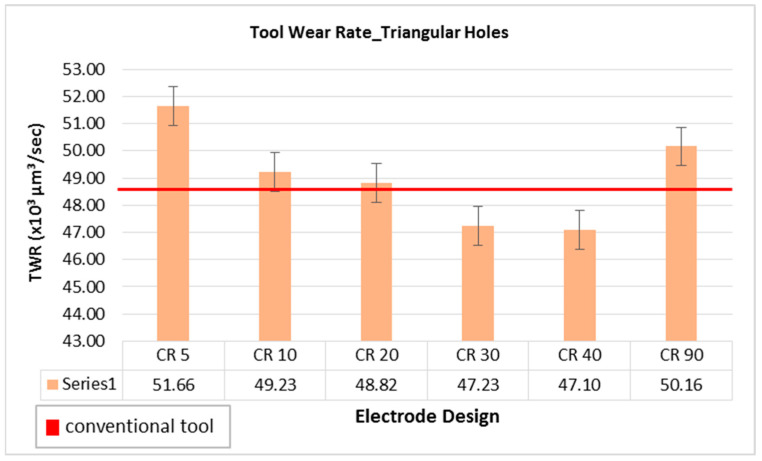
Tool wear rate while machining triangular holes with different electrode designs.

**Figure 4:**
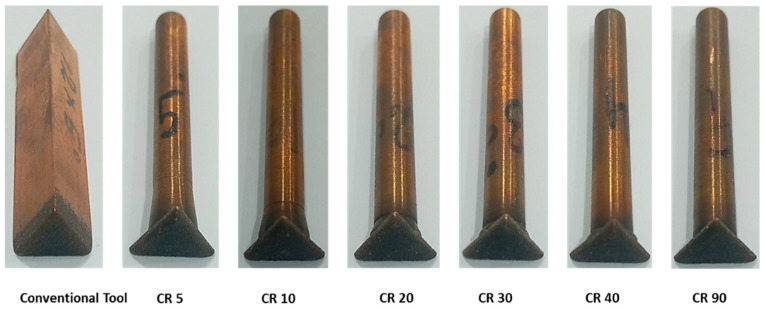
Conventional and non-conventional electrodes after machining triangular holes in D2 steel.

Considering the tool wear rate (TWR) as another important parameter of consideration in EDM die-sinking process, the comparison of TWR connected with different electrode designs has been performed, as shown in Figure 3. Figure 4 demonstrates conventional and non-conventional electrodes after machining triangular holes in D2 steel. The unit of 10^6^ µm^3^/s has been taken as a common factor of tool wear rate in all cases, as tabulated in Table 3. In this way, 48.77 × 10^6^ µm^3^/s is the value of tool wear rate in case of conventional design of electrodes. As the relief angle is increased from 5 degree to 40 degree, the tool wear continuously decreased. However, the electrode design with 90 degree relief is out of the trend, as after 40 degree the values of TWR increased abruptly (50.16 × 10^6^ µm^3^/s). It can be perceived that in electrodes with values between 50 and 80 degree relief angle, the TWR tends to increase rather than following the decreasing trend. Electrodes with relief angles less than 90 degree have three cross-sections (as shown in Figure 1) corresponding to triangular face (triangular cross-section), circular relief (continuously increasing circular cross-section), and circular shank (constant circular cross-section). It indicates that the electric current flows through the gradual changes in different cross-sections of the electrodes. On the other hand, the 90 degree angle means that there are only two segments in the tool which are triangular face and circular shank. It indicates that there is an abrupt change in the cross-section of the electrode from triangular cross-section to circular cross-section. Due to this abrupt change in the tool cross-section the electric current characteristics may become different as compared to other designs. This could be a reason behind relatively high tool wear rate in case of the 90 degree electrode.

Considering the value of 48.77 × 10^6^ µm^3^/s associated with conventional electrode as a reference of comparison, two non-conventional electrode designs (with relief angle of 30 and 40 degree, respectively) offer low amount of tool wear as compared to the wear of conventional electrode. The wear seen in the electrode design with 20 degree relief as almost the same to that of the standard electrodes. Therefore, non-conventional electrodes with 20 and 30 degree angles could be the right choice if the criteria for choosing a good electrode is to have a high MRR with a low or equivalent TWR.

**Figure 5:**
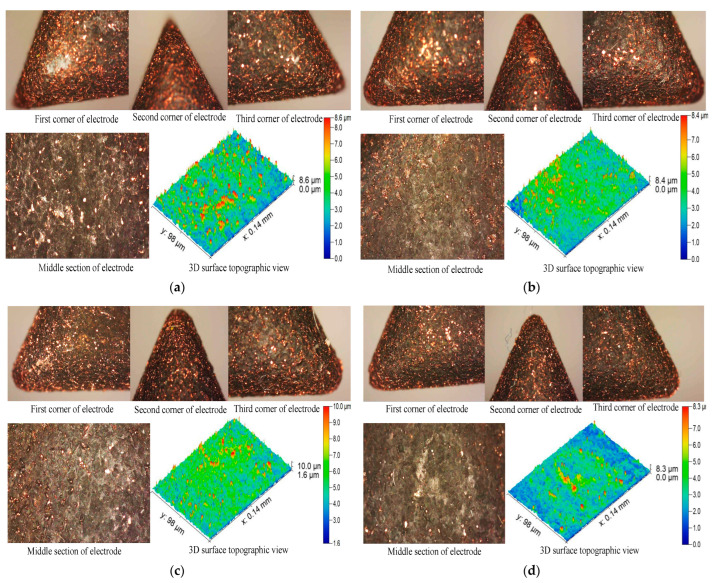
Microscopic analysis of corners, middle section and 3D surface topographic view of different electrode designs after machining: (**a**) conventional, (**b**) CR40, (**c**) CR5 and (**d**) CR90.

To demonstrate the TWR microstructural behavior of different corners, and middle sections of the machining face of conventional and non-conventional tool designs, the following Figure 5 is presented. From Figure 5, one can observe that larger, deeper and wider craters form in the case of conventional tool design (Figure 5a). From Figure 5b,c, it is clear that as the relief angle increased from 5 degree to 40 degree, the TWR decreased as the size of peaks and valleys of craters reduced.

It can be seem from Figure 5 that the tool face height reduces from 9.14 to 1 mm as the relief angle rises from 5 to 40 degrees, producing an equally powerful spark. The material is suitably eroded and the fine surface texture is produced at tool and workpiece by an equally powerful spark. The flushing mechanism may be blamed for this. The relief angle provides a steady flow path for sufficient dielectric cleaning and debris removal. Thus, when the relief angle is low (5 degrees), the path for flushing off the debris is narrow, causing some debris to interact with the tool and workpiece surface and cause irregular sparking, whereas when the relief angle is adequate (40 degrees), the flushing entrance and exit path is wider, causing negligible debris to interact with the tool and workpiece surface and re-solidify. Therefore, the corners and middle section of the CR40 electrode design wear less and very minute craters form than the conventional and other tool designs (Figure 5b). However, CR90 electrode design’s edges and middle section erode abruptly, and irregular and non-uniform craters form as compared to the CR40 (Figure 5d). The miscopy structure of the tool after machining reveals that the wear at each corner is almost uniform, as can be noticed from the very minute craters. The craters produced on the electrode surface are larger as compared to the craters at the corners. However, the crater depth seems to be almost uniform than the conventional design, indicating that the machined surface will have low surface roughness. The surface roughness achieved in each case of non-conventional electrode is discussed in the following section.

### 3.3. Effect of Tool Designs on Overcut

**Figure 6:**
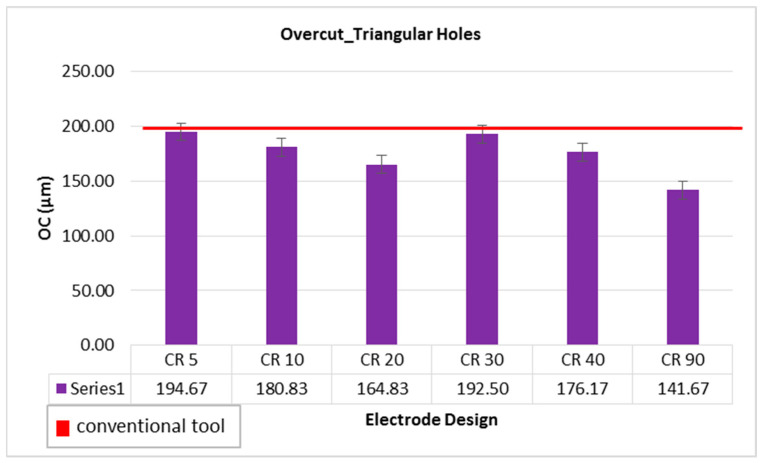
Overcut while machining triangular holes with different electrode designs.

Overcut (OC) is another important factor in EDM die-sinking as it is one of the factors determining the quality of the machined holes. The overcut is the difference between the size of the electrode face and the size of the hole. In this study, OC is measured in micrometers (µm). An overcut of 208 µm has been recorded from the hole machine by conventional electrode. Taking this value as a reference, as shown by the red line in Figure 6, it can be seen that the overcut caused by the non-conventional electrode designs is either smaller or close to the overcut of conventional design. The overcut using the 20 degree and 90 degree relief angles are ~165 µm and 142 µm, respectively. Generally, as the MRR is increased in EDM, the surface roughness and overcut values also increase. In this study, it has been found that non-conventional electrode design offers high MRR with low overcut as compared to the conventional electrode. In the same study, the relief angled designed tools have been reported as the better option to obtain less amount of overcut while machining circular holes in tungsten carbide [26]. To select the most appropriate electrode design offering high MRR, and low amounts of TWR and overcut, the electrode design with 20 degree can be considered as the common suitable choice.

**Figure 7:**
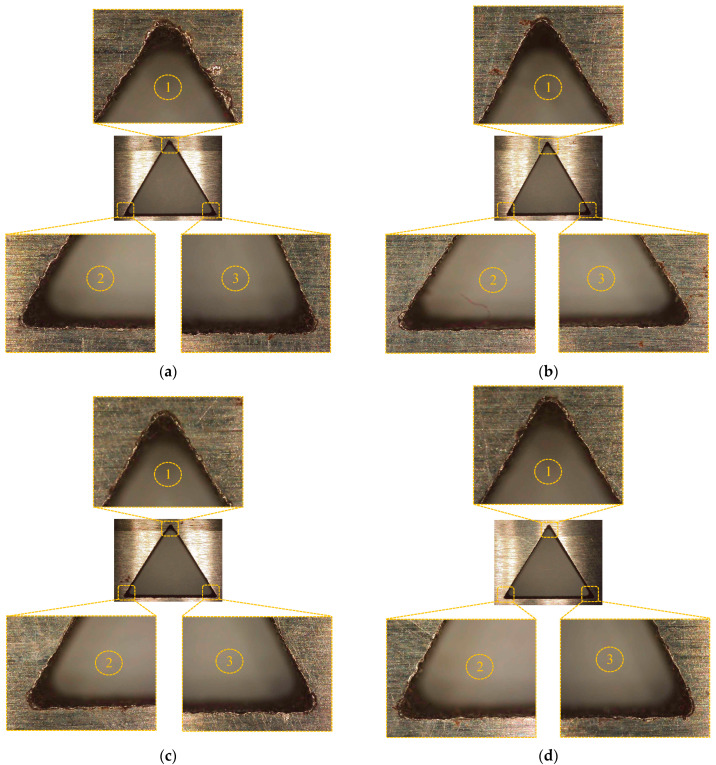
Microscopic analysis of corners of triangular holes machined with different electrode designs: (**a**) conventional, (**b**) CR90, (**c**) CR5 and (**d**) CR30. In this ①–③ indicates the magnified microscopic images of the first, second and third corner of triangular hole.

Microscopic analysis of triangular holes machined with conventional and non-conventional electrode designs has been carried out to evaluate the overcut (refer to Figure 7). Figure 7a represents the case of conventional design wherein irregularities in the hole shape, cavities, and re-deposited material have been observed at the corners of triangular hole. For the CR90 electrode, comparatively lowest overcut obtains has been found and corners of holes eroded near the net designed shape with negligible micro-cracks, as shown in Figure 7b. While for the CR5 design, maximum overcut has been obtained for CR5 and the corners of holes have been eroded in curvature shape with macro cakes, voids, and cavities instead of sharpening conical edge, as depicted in Figure 7c. Overcut dropped as the relief angle increased from 5 to 20 degrees, whereas at 30 degrees it behaved abruptly (refer to Figure 7d) and declined up to 90 degrees. Resistance in a current carrying conductor is determined by the length, cross-sectional area, material, and other considerations. As a result, the resistance in the designed electrodes is thought to change due to the varying cross-sectional areas. Consequently, the present properties may change. This might explain the uneven pattern of overcut. Resistance of conduction material is inversely proportional to cross-sectional area and has a direct relation with resistivity. In the 5 degree relief angle electrode design, length of the electrode face is high, therefore, more collision of electrode occurs as current flows and more resistance causes the random and irregular sparking phenomena.

### 3.4. Effect of Tool Designs on Hole Taper Angle

**Figure 8:**
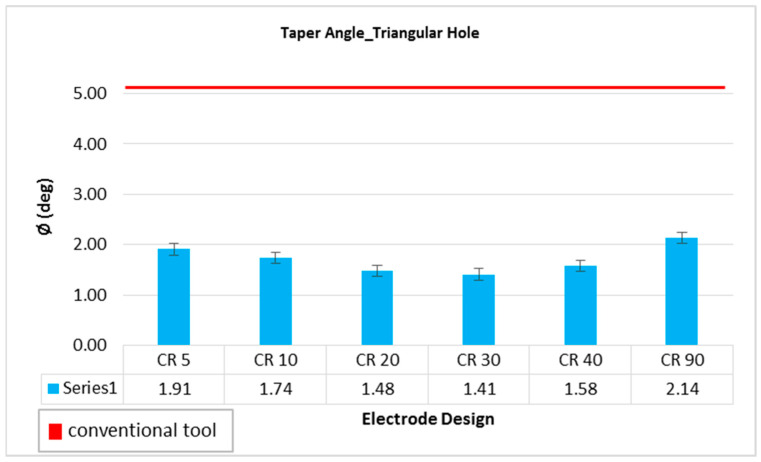
Taper angle while machining triangular holes with different electrode designs.

The quality of hole largely depends on the taper angle of the side walls of the machined holes. That is why the hole taper angle is investigated by many researchers working on EDM die-sinking. Using the conventional electrode design, a taper angle of 5.09 degree has been recorded. All the variants of non-conventional electrodes designed in this study produced significantly less amount of taper angle compared to conventional electrodes design, as depicted in Figure 8. The red horizontal line in Figure 8 represents the result of conventional design and is taken as reference for comparison. All the non-conventional electrodes produced holes with taper angle either less than or close to 2 degree. Electric discharges happen at the perimeter and at the tool-work interaction face when the usual electrode is employed. As the machining is progressed, the interacting length of the conventional design tool also increases. Thus, electric discharges are significantly more common at the electrode’s periphery as a result of this ongoing rise in the interaction region of electric sparks. This makes it harder for the molten debris to be removed from the manufacturing zone. Additionally, the molten debris contributes to re-discharging and wears down the tool’s sides. The machined hole encounters undercutting near the hole’s exit point as a result of the conventional tool’s side wall erosion, which raises the hole taper angle value. On the other hand, due of the land segment’s 2 mm height, its inclusion in the unconventional design lowers the interaction discharge area. This interacting discharge region does not continuously increase as in the case of a traditional tool as the hole development advances. Additionally, non-traditional tool designs make it easier to flush out the molten debris and lessen the side sparking that the debris causes. As a result, there is less tool wear on the side of the unconventional tool. Thus, the taper angle has been seen to decrease. Similar justification has been published in [27], which explains how the electric discharge phenomenon might occur when milling circular holes. Among non-conventional electrodes, the electrodes with 20 and 30 degree relief angles can be considered as the designs producing the holes with minimum taper, which is ~1.4 degree. A 72.5% reduction in hole taper angle can be achieved if 20 or 30 degree non-conventional electrode designs are employed to machine triangular holes in D2 steel through EDM die-sinking. High quality holes in terms of low taper angle is another advantage of the nonconventional electrodes used in this study.

**Figure 9:**
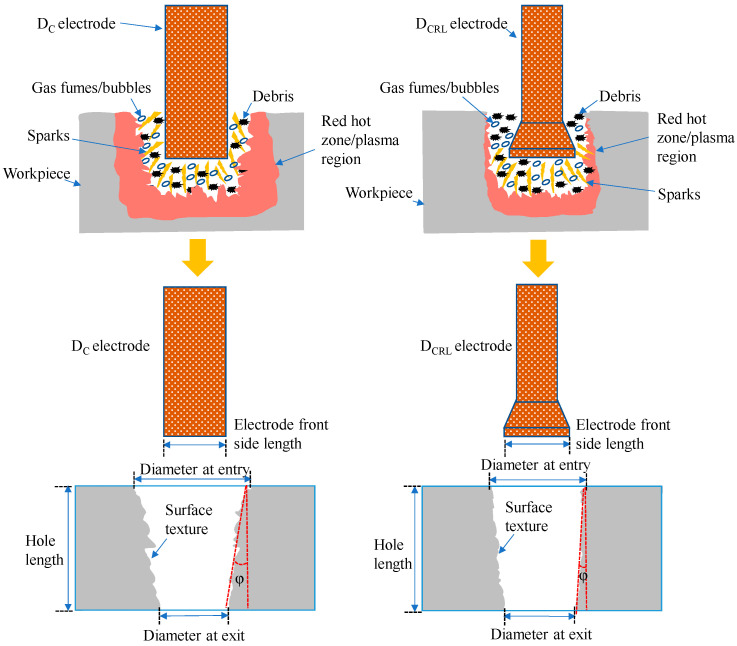
Schematic illustration of sparking phenomena during EDM and machined holes with different D_C_ and D_CRL_ electrodes.

Figure 9 illustrates the schematic of sparking processes during EDM with ordinary and non-conventional electrode configurations. It can be observed that the D_C_ design generates wider and deeper plasma region than the D_CRL_ design. The tool gradually tended to penetrate lengthwise within the machined depth in the case of the usual D_C_ design, as the machined hole’s profile advanced and a specific depth was reached. The surface area accessible to generate sparking was larger at this stage. In addition to the tool’s footing face, the side surface surrounding the tool’s periphery generated electric sparks with the machined hole’s side walls. The phenomenon is known as side sparking or peripheral sparking. During the machined feature, the surface area at the bottom of the tool remained constant. Yet, when the machining depth was raised, the surface area of the tool around the perimeter grew due to the homogeneous cross-section of the tool. As a result, the quantity of side sparking increased which causes the wider and deeper plasma region, as shown in Figure 9. The start of the machining the sparking phenomena in the D_CRL_ electrode design remained identical to standard designs. As the machining depth is increased, the sparking behavior altered. The surface area for sparkling grew up to a machining depth of 1 mm, starting with the topmost layer of the work surface (bottom surface and peripheral surface). After a machining depth of 1 mm was obtained, the increase in collective surface area (bottom and periphery) reached its maximum value. The surface area for sparking stayed constant as the depth increased since the tool’s sidewalls were straight for 1 mm and then tapered due to the availability of the relief angle. It was also observed that the quantity of side sparking grew steadily in the case of D_C_ tool design, however, it stayed constant when relief angles were incorporated into the tool design. Therefore, plasma region generated along the land surface area. Taper angle observed in the D_C_ design is larger than the D_CRL_ design, as illustrated in Figure 9.

### 3.5. Effect of Tool Designs on Surface Roughness

**Figure 10:**
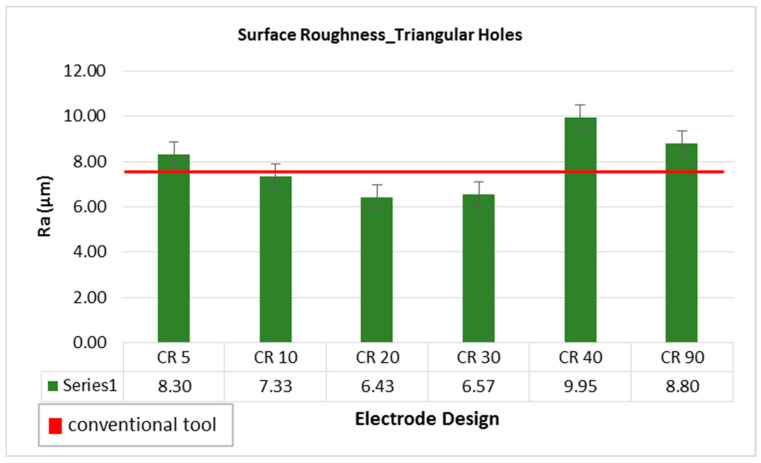
Surface roughness while machining triangular holes with different electrode designs.

A comparison of surface roughness of machined holes through different electrode designs is shown in Figure 10. The red horizontal line reflects the roughness value of R_a_ 7.43 µm corresponding to conventional electrode designs. Taking this value as a reference of comparison, it can be noticed that most of the non-conventional electrode designs offer high surface roughness in comparison with conventional design. The main reason behind this is the high MRR associated with non-conventional electrode designs as discussed earlier. However, electrodes with relief angle of 20 and 30 degree produce holes with surface roughness less than the roughness achieved through conventional design. A 13.46% improved surface finish is obtained with the CR20 design. If one has to select a certain electrode design meeting the other quality traits of the holes, the electrodes with 20 and 30 degree relief angles can be considered as the qualifying electrode designs as they have better surface roughness than the conventional design.

**Figure 11:**
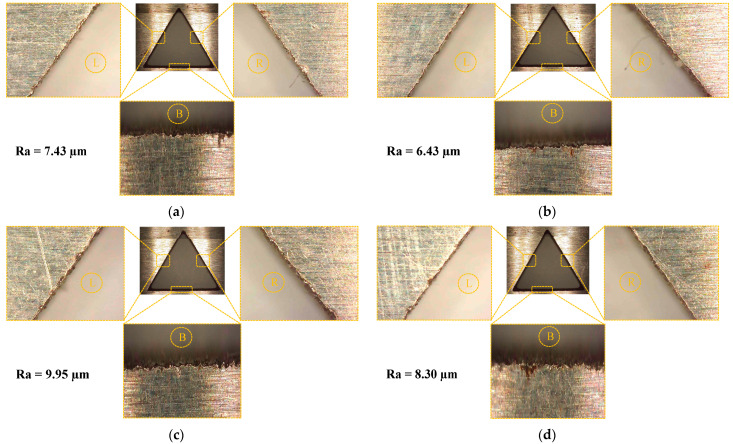
Microscopic analysis of side view of machined surface of holes with different electrode designs: (**a**) conventional, (**b**) CR20, (**c**) CR40 and (**d**) CR5.

Among non-conventional electrode designs, the electrodes with relief angles 20 and 30 degree performed better. There is close tie between 20 degree and 30 degree relief angled electrodes, particularly in terms of hole taper angle and the surface roughness. However, the electrode with 20 degree relief angle yields higher MRR and lower overcut as compared to the 30 degree electrode design. Likewise, the edge quality of the produced holes has been compared between these two designs. As the relief angle increased from 5 degree to 20 degree, the surface roughness is declined from 8.30 µm to 6.43 µm, while if the relief angle is increased above 20 degree up to 90 degree, the value of surface roughness increased. The microscopic images of the machined side surface of holes produced with conventional and non-conventional electrode designs are shown in Figure 11. In Figure 11a–d, three machined sides, (left (L), right (R) and bottom (B)), of the triangular holes are magnified. The hole corners as well as edges of the machined holes are comparatively smoother when machining is performed with 20 degree relief angle electrode design than the others, as observed in Figure 11. Particularly, the edges of the machined sides of the triangular hole produced with the CR40 electrode have unevenness.

### 3.6. Multi-Response Optimization

**Figure 12:**
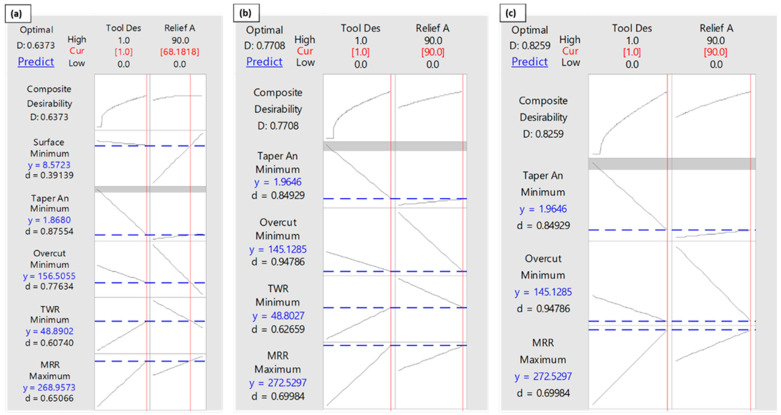
Multi-response optimization: (**a**) all five responses (Surface Roughness, Taper Angle, Overcut, TWR, and MRR), (**b**) Selected responses (Taper Angle, Overcut, TWR, and MRR), (**c**) Selected responses (Taper Angle, Overcut, and TWR).

Multi-objective optimization of EDM response measures have been evaluated using the composite desirability function (CDF) approach [28]. In this optimization, the tool designs are coded, for instance, the conventional design is coded as “0” and non-conventional design as “1”. The decision criterion of CFD is taken as the value of composite desirability close to 0.8 [29]. Considering all the five responses together (MRR, TWR, taper angle, surface roughness, and overcut), the appropriate tool achieved by this optimization is the non-conventional design with a relief angle 68 degree, as depicted in Figure 12a. However, in this case, the composite desirability remains less than 0.8. Since the surface roughness produced by non-conventional electrodes was either close to or more than the roughness caused by conventional design, therefore, the next optimization was performed by eliminating the surface roughness and considering four response measures. In this case, the composite desirability was improved and it reached to 0.77, as can be noticed in Figure 12b.

The corresponding tool is 90 degree non-conventional design resulting in optimized values of four responses. By taking three factors into consideration for optimization (taper angle, overcut and material removal rate), the achieved desirability was 0.82, as shown in Figure 12c. The optimized responses can be the hole with taper angle 1.96 degree, overcut of 145 µm and the material removal rate of 272.52 × 10^6^ µm^3^/s. The results in the form of standard error of fit, confidence interval and prediction intervals are provided in Table 4.

## 4. Conclusions

Electric discharge machining of triangular holes in D2 steel has been carried out using different non-conventional electrode designs. Material removal rate, tool wear rate, overcut, taper angle, and surface roughness are considered as the performance measures to decide the most appropriate electrode design resulting in better machining performance. Conventionally used electrode design (uniform triangular cross-section along the entire length of the electrode) is taken as the reference electrode for comparison purpose. Based on the experimental results, comparison, and percentage improvements, following conclusions can be drawn:

1. Non-conventional electrode designs perform better than the conventional electrode design for majority of the machining responses.
2. Non-conventional electrode designs significantly improve the MRR (32.76% improvement) while machining triangular holes as compared to conventional electrode design.
3. The hole taper angle produced by non-conventional electrodes is 72.5% less than the hole taper angle caused by the conventional electrode.
4. The accuracy of the machined holes (in terms of overcut) produced by non-conventional electrode designs is much better than the conventional electrode. A reduction of 20–32% in overcut can be achieved by using non-conventional electrodes.
5. Most of the non-conventional designs produce holes with surface roughness higher than the conventional electrode. However, the surface roughness by the use of 20 degree relief angle is 13.46% less than the conventional design.
6. Tool wear rate of conventional as well as non-conventional electrode designs are found closer to each other.
7. Taking into account all the performance measures (MRR, TWR, taper angle, overcut, and surface roughness), the electrode design with circular relief angle of 20 degree is the most appropriate choice to obtain the machining results close to or better than the conventional electrode design.

The future scope of the present study is to improve the machining performance measures while machining non-circular holes of other cross-sections with the help of non-conventional electrode designs employed in this study. On the other side, the limitation of the present study is the use of appropriate value of the “land height” segment of the non-conventional electrode designs. The land height should be less than the height of the hole to be produced in D2 steel.

## Figures and Tables

**Table 1:** Details of tool designs in terms of cross-sectional shape and dimensions.

Tool Design								
Conventional Designs (D_C_)		Non-Conventional Designs (Circular Relief with Land Design; D_CRL_)						
Side Length (mm)	Total Height (mm)	Design Symbol	Side (mm)	Height (mm)				Relief Angle (deg)
				Land (mm)	Face (mm)	Shank (mm)	Total (mm)	
12	50	D_CRL5_	12	2	9.14	38.86	50	5
12	50	D_CRL10_	12	2	4.53	43.47	50	10
12	50	D_CRL20_	12	2	2.2	45.6	50	20
12	50	D_CRL30_	12	2	1.4	46.6	50	30
12	50	D_CRL40_	12	2	1	47	50	40
12	50	D_CRL90_	12	2	0	48	50	90

**Table 2:** EDM parameters and their values used in machining D2 steel.

**Hole and Workpeice Parameters**						
Hole Type	Hole Shape		Hole Depth (mm)	Workpiece Material	Workpiece Thickness (mm)	
Through Hole	Triangular		4	AISI D2 Steel	4	
**Tool Parameters**						
Tool Material	Tool Height (mm)		Shank Cross-Section	Shank Diameter (mm)	Face Cross-Section	Face Length (mm)
Copper	50		Circular	6	Triangular	12
**Machining Parameters**						
Discharge Current (A)	Spark Voltage (V)	Pulse On-Time (µs)	Pulse Off-Time (µs)	Spark Time (s)	Flush Time (s)	Dielectric
20	5	100	50	5	5	Kerosene oil

**Table 3:** Experimental results in terms of various machining responses.

Tool Designs		Material Removal Rate; MRR (×10^6^ µm^3^/s)	Tool Wear Rate; TWR (×10^6^ µm^3^/s)	Overcut; OC (µm)	Taper angle; φ (deg)	Surface Roughness; R_a_ (µm)
Conventional	C0	221.70	48.77	208.00	5.09	7.43
Circular Relief	CR 5	293.62	51.66	194.67	1.91	8.30
	CR 10	274.82	49.23	180.83	1.74	7.33
	CR 20	251.61	48.82	164.83	1.48	6.43
	CR 30	221.93	47.23	192.50	1.41	6.57
	CR 40	242.38	47.10	176.17	1.58	9.95
	CR 90	294.33	50.16	141.67	2.14	8.80

**Table 4:** Multi-response optimization results for different cases of response groups.

Tool Design	Relief Angle	Composite Desirability	Response	Fit	SE Fit	95% CI	95% PI
Multi-response optimization—All five responses (Surface Roughness, Taper Angle, Overcut, TWR, and MRR)							
1	68.18	0.63	Surface Roughness (µm)	8.57	0.91	(6.0, 11.1)	(3.9, 13.2)
			Taper Angle (deg)	1.86	0.17	(1.3, 2.3)	(0.9, 2.7)
			Overcut (µm)	156.51	8.33	(133.3, 179.6)	(114.4, 198.5)
			TWR (×10^6^ µm^3^/s)	48.89	1.28	(45.3, 52.4)	(42.4, 55.3)
			MRR (×10^6^ µm^3^/s)	269.0	21.2	(210, 327.9)	(161.6, 376.3)
Multi-response optimization (Selected responses) (Taper Angle, Overcut, TWR, and MRR)							
1	90	0.77	Taper Angle (deg)	1.96	0.25	(1.3, 2.6)	(0.9, 2.9)
			Overcut (µm)	145.1	11.7	(112.6, 177.7)	(97.2, 193.0)
			TWR (×10^6^ µm^3^/s)	48.8	1.8	(43.8, 53.8)	(41.4, 56.17)
			MRR (×10^6^ µm^3^/s)	272.5	29.9	(189.5, 355.6)	(150.3, 394.7)
Multi-response optimization (Selected responses) (Taper Angle, Overcut, and TWR)							
1	90	0.82	Taper Angle (deg)	1.96	0.25	(1.3, 2.6)	(0.94, 2.98)
			Overcut (µm)	145.1	11.7	(112.6, 177.7)	(97.2, 193.0)
			TWR (×10^6^ µm^3^/s)	48.80	1.80	(43.8, 53.8)	(41.4, 56.1)

## Data Availability

Data are contained within this article.
